# Supplementary material for: Acceptability and feasibility of tests for infection, serological testing, and photography to define need for interventions against trachoma
Source: PLoS Negl Trop Dis. 2024 Jun 6;18(6):e0011941. doi: 10.1371/journal.pntd.0011941 (PMC11185441; doi:10.1371/journal.pntd.0011941)
Supplement: S2 File — (PDF) [file pntd.0011941.s002.pdf]

Tanzania Alternative Indicators

Codes

Name	Description
Blood advantages	Advantages to swabs (compared to other test types)
Blood disadvantages	Disadvantages of blood spots (compared to other test types)
Blood experience	Participant has experience with blood spot
Blood would not participate	If participant would not participate in blood spot
Blood would participate	If participant would participate in blood spot
Photo advantages	Advantages to photography (compared to other test types)
Photo disadvantages	Disadvantages of photography (compared to other test types)
Photo experience	Whether or not participants have experience with conj. photography. (Positive/negative experiences will be coded under advantages/disadvantages.)
Photo would not participate	If participant would not participate in photography
Photo would participate	If participant would participate in photography
Swab advantages	Advantages to swabs (compared to other test types)
Swab disadvantages	Disadvantages of swabs (compared to other test types)
Swab experience	Participant has experience with eye swab

Name	Description
Swab would not participate	If participant would not participate in eye swab
Swab would participate	If participant would participate in eye swab
test ranking	Direct comparison of test types to each other (sentiments like "better","worse", "I would prefer", etc.)
TF advantages	Advantages to TF grading (compared to other test types)
TF disadvantages	Disadvantages of TF grading (compared to other test types)
TF experience	Whether or not participants have experience with TF grading. (Positive/negative experiences will be coded under advantages/disadvantages.)
TF would not participate	If participant would not participate in TF grading.
TF would participate	If participant would participate in TF grading.
