# Supplementary material for: Acceptability and feasibility of tests for infection, serological testing, and photography to define need for interventions against trachoma
Source: PLoS Negl Trop Dis. 2024 Jun 6;18(6):e0011941. doi: 10.1371/journal.pntd.0011941 (PMC11185441; doi:10.1371/journal.pntd.0011941)
Supplement: S5 File — (PDF) [file pntd.0011941.s005.pdf]

Category	Key questions
Setting/interpreting meaningful thresholds	What is the threshold for infection? How does this vary in different settings? Will the indicator be presence/absence of *Chlamydia trachomatis (Ct)* , or will typing occur to determine serotype?
	What is the threshold for serology? How does this vary in different settings? How do age dynamics affect thresholds/cutoffs?
	What is the average change in trachomatous inflammation--follicular (TF) prevalence from trachoma impact survey (TIS) to trachoma surveillance survey (TSS)?
	Is the 5% prevalence threshold for TF in children aged 1-9 years adequate to ensure that trachoma will not spread in the community?
	How can we trust/validate any thresholds?
	Can community-level (vs. other-level) thresholds be set? How to understand infection results at community
Test characterstics (general)	What is the sensitivity/specificity/positive predictive value (PPV)/negative predictive value (NPV) of each test?
Test characterstics (photography)	How accurate is human grading compared to grading via photography with artificial intelligence (AI)? Does this differ by setting?
	How to interpret any increased sensitivity of photography compared to field grading of TF? Should we consider follicles outside the inclusion zone or <0.5mm?
Survey design/screening protocols	Can we develop models for integrating other indicators (including other clinical signs) into trachoma screening to accurately estimate infection?
	Can other indicators be used to confirm a TF diagnosis?
	Has the current method of pooling been validated? What's the best number of samples to pool to bring down cost/time of analysis without a loss in accuracy?
	What infection testing platform would be best: multiplex PCR or could a point-of-care (POC) test be useful?
	What's the best age group to test?
	How many people need to be tested? Does this differ by TF prevalence setting?
	What testing protocol produces best sensitivity/specificity/PPV/NPV?
Interpreting serological data	How to interpret different sero- and sub-types of Chlamydia? (ex: ocular vs. STI)
	How to interpret role of seroreversion?
	What is the relationship between seroconversion due to ocular exposure vs. other routes?
	What is the load or frequency of infection required to cause seroconversion?
	How do we test for/interpret previous exposure?
Clinical signs/progression of disease	What is the duration of clinical signs in areas with formerly high levels of trachoma?
	What is the temporal variability of infection (i.e. how much does exact timing of swab collection matter vs. TF, which is positive for longer and so timing matters less)?
	How does infection relate to fine-graded TF in an individual? (All we have is pooled/community level data.)

Category	Key questions
	Why is there a lag between clearance of infection and resolution of TF?
	What is the progression from infection to TF?
	In settings with a formerly high level of trachoma, are clinical signs indicative of scarring?
	In surveillance settings, are clinical signs sufficiently rapid and sensitive to detect infections that will lead to later scarring/trachomatous trichiasis (TT)?
	What evidence supports the number, size, and position of follicles in the definition of TF?
	Should resolving follicles (from children treated with azithromycin) be included in the calculation of TF prevalence?
	How to differentiate dead follicles from active ones?
	What's the longitudinal trajectory of infection?
	What is the relationship between seropositivity and continued scarring progression once elimination thresholds have been reached?
Integration within existing systems	How to integrate infection testing into ongoing surveys done by national programmes?
	Can TF grading be incorporated into primary care/routine child health care/school-based programs to be used effectively as post-elimination surveillance to detect potential recrudescence
	How can infection testing be integrated with other neglected tropical disease (NTD) testing?
	How to integrate photography with other data collection platforms?
	Can we certify graders using Tropical Data (TD) system for all eye health care workers to routinely conduct TF grading?
Feasibility/acceptability issues	What are the ethical considerations of photography (concerns about anonymity) and will this increase training time/survey time and therefore cost?
	What are the costs compared to other indicators?
	Are photos acceptable to communities?
	How can AI be used (with photography) and what is the cost of this?
	How can photography be used at scale?
	Is infection testing acceptable to communities?
	What's the cost at-scale and how can the cost be reduced by using regional testing centers?
	How to handle increased logistics needs for infection testing?
	What's the additional costs associated with sample management vs. the sampling itself?
	What is the community acceptability of serology?
	How to ensure adequate funding?
	How to ensure trust with communities that samples will be used only for trachoma testing?

Category	Key questions
	What will the cost reduction be by not doing mass drug administration based (only) on TF results?
	What are the actual relative costs of TF, photography, eye swabs, serology? What does "increased costs" actually signify?
	How much, for each indicator, is there capacity in-country, and how much are countries reliant on external partners?
Mitigating training/lab quality issues	How to mitigate the loss of skill of TF grading in low-prevalence/post-elimination settings?
	How can we standardize training and grading?
	How to ensure quality photos?
	How to build lab capacity in country or regional settings?
	How to train people adequately?
	Which camera equipment best?
	How to upload/share quality photos?
	How to ensure consistency in photography use/specifications/training, etc.?
	How to monitor for lab contamination?
	How to ensure safety of community members/training of swab-takers?
	How to ensure adequate lab capacity/training to ensure quality samples/standardized analysis?
	How to mitigate the difficulty and inherent subjectivity of grading/interpreting results in low-prev settings?
	How long does it take on average from sample collection to results generation across multiple settings?
	What's the stability of swabs in different settings and time to arrival to lab?
other	Is DNA detection good enough, or should we be looking for viable organisms?
	Why is there recrudescence of TF in the absence of infection/other signs?
	How to ensure development/roll out of a rapid diagnostic test?
