# Supplementary material for: Acceptability and feasibility of tests for infection, serological testing, and photography to define need for interventions against trachoma
Source: PLoS Negl Trop Dis. 2024 Jun 6;18(6):e0011941. doi: 10.1371/journal.pntd.0011941 (PMC11185441; doi:10.1371/journal.pntd.0011941)
Supplement: Attachment — Submitted filename: response to reviewers - Accept Feas Trachoma.docx [file pntd.0011941.s006.docx]

Thank you so much for reviewing this manuscript and providing feedback. Relevant comments are below, with our responses in red.

In addition to the revisions based on your feedback detailed below, we've also reported 5-year age bins, rather than specific ages, in the attributions of the quotations from focus group members. This was a change requested by MedRxiv prior to publication on their pre-print server.

Reviewer #1: Minor change, in the first paragraph of discussion, the authors refer to the IDI respondents as "health workers," but I would recommend they continue to use public health practitioners, as "health worker" sounds similar to "healthcare worker" and this was only 1 of 11 of this group.

Updated; thanks!

Reviewer #1:
My main comments center around the utility of answers from the focus groups which seem to be based on inaccurate on contradictory assumptions. For example, the respondents voiced support of photography since it could be quicker than in person exam, but I am not sure that this is true, as the lid must still be everted and then the correct exposure must be acquired. Likewise, a major concern about photography seemed to be the flash, but not all programs have used a flash. The authors indicate that the timing of results (ie delayed and not directly communicated to individuals) was not always conveyed to focus group participants, but whether the authors believe this would have a major effect on their inferences is not completely explored. Finally, assumptions about the accuracy and modernity/legitimacy about infection/serology testing do not seem completely accurate as discussed by focus group participants. The authors indicate that these tests are complementary, but there seems
to be an implied preference for one test over another, rather than people being exposed to a possible battery of tests.

Overall, with all of this, I think a little more treatment in the discussion about the role of (gauging/setting) expectations of a given new modality would be helpful, as it seems clear that positive sentiments (ie about the legitimacy of medical tests) might benefit an initial survey, but if other expectations are not met (about the immediacy of test results), this could severely hamper subsequent surveys.

Thank you for these comments! The paragraph in the limitations section starting on line 572 has been edited to address this. We also note that understanding common misconceptions is also important, and will help programs target sensitization efforts accordingly.

Also, the advantage of monitoring for other diseases is an advantage of the medical tests, which the authors imply was under-appreciated by respondents. It is unclear how to balance this advantage with legitimate concerns about the samples being used to detect unrelated diseases (e.g., HIV). How would the authors recommend balancing this in a program?

We believe that it is up to country programs to find this balance, given the cultural preferences in their own contexts! We added a few sentences encouraging programs to consider this and underscoring the need for ethical approval and participant consent for any future testing. (see lines 520 to 524)

Reviewer #3: A mixed methods study about the acceptability of conjunctival photography, conjunctival swabbing, and serology for trachoma surveillance. Overall I found this to be well designed and well reported. I just have a couple very minor comments:

Table 2 was cut off on the right hand side

I saved the file in "draft view" should resolve this issue. Alternatively, you can download the .docx file and open it in draft view to see the full table.

**Line 433:** minor comment but the mention of “agreement” made me think this analysis was assessing some measure of inter-respondent agreement. But here I think this is simply a stratified analysis? I might word it a little differently, saying that on average the country staff identified conjunctival photography to face the greatest barriers to community acceptability, and that in contrast, the other stakeholders identified serology as facing the greatest barriers

Thanks for the feedback! You are correct that we did not measure inter-respondent agreement, but rather a stratified analysis comparing the level of agreement of different groups that the various statements on barriers were true. So while "level of agreement" is not necessarily incorrect, using this term less often should hopefully preclude confusion. This has been addressed in the manuscript by rewording this section (starting line 439).

A minor comment: there have been many trachoma studies conducted throughout the world that have incorporated conjunctival photography, conjunctival swabs, and serology, some of which have sampled the same participants longitudinally over years. I am not aware of massive refusals of any of these testing modalities in these studies, which also lends some support for the idea that all three of these methods are acceptable to community members.

We agree that anecdotally, refusals do seem rare. Unfortunately, it's also rare for refusal rates to be reported! We see this as an important addition to the introduction, as it highlights the need for a better understanding of the acceptability of the test types in a variety of contexts. (See lines 110 to 114.)
